# Treatment of Chronic Metritis

**Published:** 1867-01

**Authors:** 


*Treatment of Chronic Metritis.*—Remarks by Prof. Baker before the New York Academy of Medicine:—

Dr. Bennett is entitled to the merit of being the first English author, who in a sound, pathological manner, called the attention of the profession to the existence of this disease. Since then other writers have presented their views on this subject, but they were nearly a reproduction of the views of Bennett. The treatment which Bennett regarded as most successful, was the establishment of an issue on the cervix uteri, for the purpose of draining off the inflammatory exudative deposit which had taken place in the body of the uterus; and modifying the increased inflammatory action which each menstrual period produced. His favorite agents were the potassa cum calce, or potassa fusa, for producing a permanent issue in the cervix. In relation to both of these measures, he himself had not the confidence with which they seemed to be generally regarded. Fifteen or twenty years ago, he was in the habit of constantly and frequently resorting to depletion, either by leeches, or direct scarification of the cervix uteri. While he agreed with Dr. Peaslee that there is a marked reduction of the symptoms, if nota palliation, especially if practised just before the menstrual period; yet he has found there was no real permanent good, but, after successive operations, the patients, instead of getting ahead, were retrograding. He believed also, that in the large majority of these cases of chronic engorgement, or inflammation, the patient is in a condition of anæmia. This is true in almost all cases. The depletion is not directly to the organ, but to the cervix uteri, and whether the amount of liquor sanguinis of the body of the uterus becomes decreased thereby, seems, I think, very doubtful. He had satisfied himself that patients are injured by this method of treatment. There are conditions when he used leeches, but chronic metritis is not one of these conditions. In relation to the constitutional treatment, he would not enter into a full discussion.

"In regard to the regimen and hygienic measures, which are important and necessary, he agreed with Dr. Peaslee fully. In relation to mercurials, he agreed with both him and Dr. Budd. Instead of the bichloride, he was in the habit of using the protoiodide of mercury, in two-grain doses, combined with the sulphate of iron, and with a small quantity of opium, or some of its preparations, both to allay nervous irritation, and check any tendency of the protoiodide to irritate. But there are two methods of treatment to which he wished especially to call attention, and which had seemed, in his practice, of great value. One is the use of large injections of hot water into the pelvic cavity. This is especially for that form of chronic metritis associated with induration; and associated ordinarily with dysmenorrhœa, sometimes amenorrhœa, or menorrhagia. The effect of the long-continued application of hot water on the tissues can be easily ascertained, and is perfectly familiar to almost every person present, and can be shown by holding the hand in it for any length of time. It has the effect of liquifying, so to speak, the plastic exudation, and producing absorption and resolution. It is now four or five years since he had been accustomed to using it in this special form of chronic metritis. A very large quantity should be used each time; the patient should be placed on the bed, in precisely the forceps position; an India rubber sheet is placed under her; a sheet is placed over her, so that there is not the slightest exposure. Under this rubber sheet a pail of water is placed, at a temperature as high as the patient will tolerate. The patients will bear a degree of temperature much hotter when injected into the vagina than they can bear by immersion of the hand. A Davidson's injecting syringe, one end of the tube dropping into the pail of water, the other in the vagina, injects the water into the vagina, which runs directly back into the pail. There is no need of wetting the clothes. You can add hot water as it is necessary.

"The patient must be shown how to make the application in this way. Several gallons of water are injected at a time, and the injection continued fifteen or twenty minutes. As regards the success of this treatment, he had arrived at results with it more satisfactory than from any other mode of treatment. Those who would try this should particularly notice the extraordinary change the tissue undergoes in the whole vaginal wall after the water has been introduced for a long time. Some may have used this for the induction of premature labor. The change which is immediately produced in the tissues is astonishing. In that form of chronic metritis associated with amenorrhœa, and especially dymenorrhœa, be restored to this treatment daily for four or five days previous to the appearance of each menstrual period; and often at the very first period succeeding its use, great relief is experienced. It is accompied with great modification of dysmenorrhœal pains, and great increas of the menstual dischange. In this way we have a normal, natural method of directly depleting the organ, which favorresolution and absorption of the antecedent inflammatory des posit."

